# A Case of Drug-Induced Pancytopenia due to Tamoxifen

**DOI:** 10.70352/scrj.cr.25-0227

**Published:** 2025-07-31

**Authors:** Akari Takahashi, Saori Fujiwara, Yui Takahashi, Maya Isoda, Mio Yasukawa, Kyoko Goda, Takashi Yamanaka, Toshinari Yamashita, Shu Yuguchi

**Affiliations:** 1 Kanagawa Cancer Center Department of Breast Surgery and Oncology, Yokohama, Kanagawa, Japan; 2 Kanagawa Cancer Center Department of Pathology, Yokohama, Kanagawa, Japan

**Keywords:** tamoxifen, cytopenia, pancytopenia

## Abstract

**INTRODUCTION:**

Tamoxifen (TAM) is a well-established treatment for hormone receptor-positive breast cancer with a known side-effect profile that includes hot flashes, genital bleeding, and diarrhea (0.1%–5%). Other notable side effects include liver damage, abnormal vaginal discharge, depression, dizziness, and headaches of unknown frequency. However, blood cell count reduction has not yet been reported as a side effect in Japan.

**CASE PRESENTATION:**

A 46-year-old female patient was diagnosed with right breast cancer (cT1N0M0). The patient underwent partial right breast resection and sentinel lymph node biopsy. Owing to the positive surgical resection margin, a mastectomy was performed. Pathological analysis of the surgical specimen confirmed invasive ductal carcinoma (estrogen receptor [ER]: 95%, progesterone receptor [PgR]: 85%, HER2: 2+ [fluorescence *in situ* hybridization, FISH negative]), with macrometastasis in one sentinel lymph node. Postoperative treatment included chemotherapy (dose-dense adriamycin and cyclophosphamide [AC] to dose-dense paclitaxel [PTX]), irradiation, and TAM. While initial blood test results before starting TAM showed mild anemia (Hb: 8.9 g/dL Grade 2), a follow-up blood test 5 months after initiating TAM revealed a significant decrease in blood cell counts (white blood cell [WBC]: 2600/μL Grade 2, neutrophil [neu]: 0.55 × 10³/μL Grade 3, Hb: 7.7 g/dL Grade 2, platelet [PLT]: 13.3 × 10⁴/μL). Considering the onset of symptoms following TAM administration, drug-induced pancytopenia was suspected. TAM and its concomitant medication pregabalin were discontinued. However, the blood cell counts continued to decline, necessitating further investigation. Myelodysplastic syndrome (MDS) was suspected, leading to multiple bone marrow biopsies. However, no definitive hematological disorder was diagnosed. The patient received transfusions and granulocyte colony-stimulating factor (G-CSF) injections based on the blood cell count. Approximately 4 months after the onset of neutropenia, gradual recovery was observed and spontaneous remission occurred. Given the rarity of spontaneous recovery from MDS, TAM is considered a potential causative agent of the observed decline in blood cell counts.

**CONCLUSIONS:**

We report a case of suspected drug-induced cytopenia associated with tamoxifen administration.

## Abbreviations

AC
: adriamycin and cyclophosphamide
DCIS
: ductal carcinoma *in situ*
DFS
: disease-free survival
ER
: estrogen receptor
EXE
: exemestane
FISH
: fluorescence *in situ* hybridization
G-CSF
: granulocyte colony-stimulating factor
Hb
: hemoglobin
HG
: historical grade
IDC
: invasive ductal carcinoma
LH-RHa
: luteinizing hormone-releasing hormone agonist
MDS
: myelodysplastic syndrome
MRI
: magnetic resonance imaging
Neu
: neutrophil
NG
: nuclear grade
OS
: overall survival
PgR
: progesterone receptor
PLT
: platelet
PTX
: paclitaxel
RBC
: red blood cell
STIR
: short tau inversion recovery
TAM
: tamoxifen
WBC
: white blood cell

## INTRODUCTION

TAM is widely used to treat hormone receptor-positive breast cancer. Postoperative TAM administration significantly improved DFS (hazard ratio [HR]: 0.64) and OS (HR: 0.89). It has also been shown to reduce breast cancer-related mortality (mortality ratio: 0.71), with benefits lasting for at least 10 years after surgery.^1)^

Common TAM-related adverse effects include nausea and vomiting (>5% incidence), hot flashes, genital bleeding, and diarrhea (0.1%–5% incidence). Other adverse effects of unknown frequency include liver damage, abnormal vaginal discharge, depression, dizziness, and headaches. Serious but rare side effects include agranulocytosis, blood cell count reduction, thrombosis, uterine fibroids, and endometriosis.^2,3)^ In women aged 55 and older, TAM use has been associated with a 3.8% increased risk of endometrial cancer, whereas no increased risk was observed in younger women.^1)^

Despite blood cell count reduction being a known serious side effect, no cases have been reported in Japan, and only a few cases have been documented internationally.^4,5)^ This report describes a case of severe TAM-induced pancytopenia.

## CASE PRESENTATION

A 46-year-old woman with no significant allergies, or medical or family history was diagnosed with cT1N0M0 stage I IDC.

### Diagnosis and Initial Treatment

The patient was preoperatively diagnosed with IDC, showing 90% ER positivity, 90% PgR positivity, and HER2 expression at 2+, with FISH confirming a negative result. Pathological analysis confirmed IDC historical grade (HG)II nuclear grade (NG)1, with an invasive tumor measuring 10 × 9 × 9 mm. ER expression was 95%, PgR expression was 85%, and HER2 remained at 2+ (FISH-negative), with a Ki67 index ranging from 5% to 20%. The surgical margin contained DCIS, and one out of three sentinel lymph nodes exhibited a 3.5 mm macrometastasis. Consequently, the patient underwent a mastectomy and axillary radiation therapy. The final pathology confirmed DCIS with a tumor size of 90 × 43 × 7 mm, leading to the final diagnosis of pT1cN1aM0 stage IIB breast cancer.

**Fig. 1:**
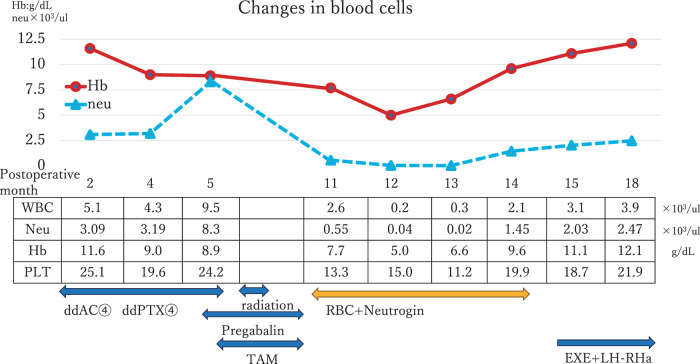
Clinical course of patients from postoperative blood cell recovery. ddAC, dose-dense adriamycin and cyclophosphamide; ddPTX, dose-dense paclitaxel; EXE, exemestane; Hb, hemoglobin; LH-RHa, luteinizing hormone-releasing hormone agonist; Neu, neutrophil; PLT, platelet; RBC, red blood cell; TAM, tamoxifen; WBC, white blood cell

The postoperative treatment plan included chemotherapy with dose-dense AC for 4 cycles, followed by dose-dense PTX for 4 cycles. The treatment period was 4 months, with administration every 2 weeks and subcutaneous injection of Pegfilgrastim 2 days after each administration. This was followed by radiotherapy at 50 Gy over 25 fractions, and subsequent hormone therapy with tamoxifen. During chemotherapy, the patient developed grade 2 peripheral neuropathy, leading to the prescription of pregabalin (at 75 mg twice daily). Upon completion of chemotherapy, routine blood tests revealed mild anemia with a Hb level of 8.9 g/dL (Grade 2) but no significant hematologic abnormalities (**Fig. 1**).

**Fig. 2:**
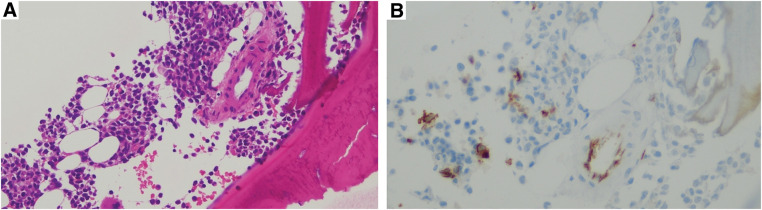
Although immature cells were observed (**A**), no CD34+ (**B**) blasts were seen, and there were no findings suggestive of breast cancer bone metastasis, myelodysplastic syndrome (MDS), or leukemia.

After 5 months of starting TAM and 11 months post-surgery, blood tests showed a progressive decline in blood cell counts. The white blood cell (WBC) count dropped to 2600/μL (Grade 2), with neutrophils at 570/μL (Grade 3). Hemoglobin further decreased to 7.7 g/dL (Grade 3), and platelets measured 19.4 × 10^4^/μL. A bone marrow biopsy revealed the presence of immature cells but no CD34+ blasts (**Fig. 2**), ruling out the possibility of breast cancer metastasis, MDS, or leukemia. Iron and vitamin B12 levels were within normal ranges, and flow cytometry did not indicate dysplasia or blast proliferation.

**Fig. 3:**
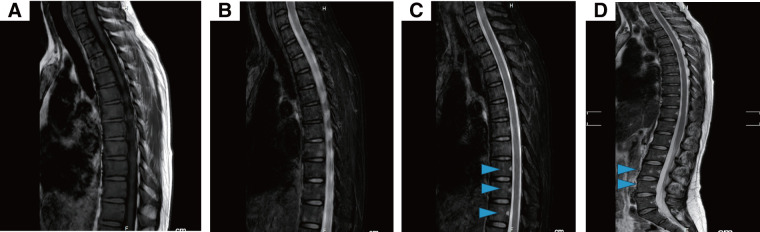
In typical cases of aplastic anemia, fatty marrowization causes a uniform high signal on T1-weighted images. In this case, a diffuse low signal was seen on T1 (**A**), and on T2 (**B**) and STIR (**C**, **D**), the distribution of high signal areas in the spinal cord did not follow the normal pattern, and an uneven pattern was seen (arrowheads). Instead of the fatty marrow seen in aplastic anemia, scattered cellular marrow was seen. STIR, short tau inversion recovery

**Fig. 4:**
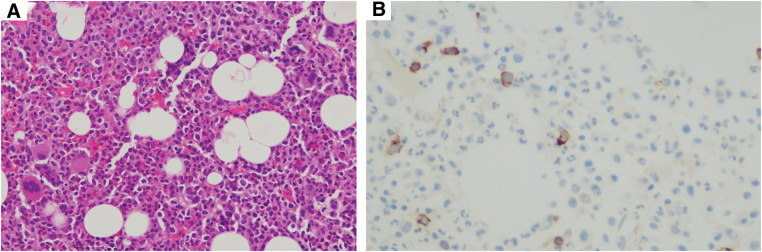
Approximately 5% of the blasts (**A**) were CD34+ (**B**), and the possibility of myelodysplastic syndrome (MDS) remained, but the possibility of the effect of G-CSF was high. G-CSF, granulocyte colony-stimulating factor; MDS, myelodysplastic syndrome

Given the suspected drug-induced nature of the cytopenia, TAM and pregabalin were discontinued. Despite this, blood cell counts continued to decline, the WBC count reaching 500/μL (Grade 4), with Neus at 130/μL (Grade 4) and Hb 5.8 g/dL (Grade 3), accompanied by symptoms of dizziness and fatigue. Thoracolumbar MRI revealed no evidence of fatty marrow (**Fig. 3**), excluding aplastic anemia. Repeated bone marrow biopsies (**Fig. 4**) and flow cytometry failed to provide a definitive diagnosis. The patient was admitted to a clean room, and received transfusions and Neutrogin; however, immediate recovery was not observed.

Two months after the onset of cytopenia, hematopoietic stem cell transplantation was considered. However, blood cell counts spontaneously recovered (**Fig. 1**), which is rare in patients with MDS. By 15 months post-surgery, the blood cell counts had normalized, allowing the patient to begin treatment with exemestane and a luteinizing hormone-releasing hormone agonist. No recurrence of cytopenia have been reported since then, to date.

## DISCUSSION

The frequency and onset of TAM-induced blood abnormalities remain unclear. Consequently, there are no established guidelines on the necessity or timing of testing. According to the National Cancer Institute’s Dictionary of Cancer Terms, pancytopenia refers to “a condition in which there is a lower-than-normal number of red and white blood cells and platelets in the blood”.^6)^ This case was diagnosed as pancytopenia because it met the following criteria WBC (3300/μL), Hb (11.5 g/dL), and PLT (15 × 10^4^/μL) or less.

Potential causes of blood cell count reduction include hematologic malignancies, such as leukemia, myelodysplasia, congenital disorders, drug-induced effects, and radiation therapy. However, further examination ruled out these potential etiologies in this case. Spinal MRI, a valuable tool for diagnosing aplastic anemia, was performed in this case. According to the reference guide for the treatment of aplastic anemia, the typical MRI findings of aplastic anemia show a uniformly high signal on T1-weighted images because of fatty marrow replacement. To accurately assess hematopoietic function, fat-suppressed images should be evaluated alongside standard MRI scans.

Kusumoto et al.^7)^ proposed the classification of bone marrow hematopoietic function using STIR images, dividing the findings into four types: type 1, characterized by very few high-signal areas; type 2, a normal pattern with high-signal areas around the vertebrae; type 3, an uneven distribution of high-signal areas deviating from the normal pattern; and type 4, an increased distribution of high-signal areas. Type 1 typically indicates fatty marrow, while type 4 is associated with cellular marrow. Severe aplastic anemia frequently presents as type 1, whereas MDS often occurs as types 3 and 4. However, hypoplastic MDS may exhibit type 1, and many cases of moderate aplastic anemia manifest as type 3, thus complicating the differentiation between the two conditions on MRI.^7)^

In this case, the MRI findings were consistent with the description of type 3, according to the reference guide for aplastic anemia treatment. Based on the flow cytometry results and the spontaneous improvement of symptoms after drug discontinuation, the condition was determined to be drug-induced rather than a primary hematologic disorder.

Based on the clinical course, the drugs suspected to cause cytopenia in this case were tamoxifen and pregabalin. Although pregabalin had been initiated 1 month prior to TAM, the patient did not exhibit any hematologic symptoms during pregabalin monotherapy. Easy bruising and signs of anemia began to appear only after the initiation of TAM. Moreover, while pregabalin has been associated with leukopenia, neutropenia (0.3%–1%), and thrombocytopenia (<0.3%), no cases of anemia have been reported.^8)^ Cases of agranulocytosis reported with pregabalin did not involve anemia or thrombocytopenia either.^9)^ These findings suggest that TAM is the most likely causative agent of the observed pancytopenia. However, a limitation of this case is that it remains difficult to definitively determine which drug was responsible for the hematological toxicity. Since both TAM and pregabalin are potential causative agents, and considering the ethical constraints, readministration of the suspected drug to confirm causality is not feasible. Thus, the diagnosis must rely on clinical judgment and the exclusion of other possible causes.

Hematological toxicity is a well-documented complication of chemotherapy and other related agents. A review of the PubMed and Ichushi databases identified several case reports describing TAM-induced agranulocytosis and leukopenia,^4,5,10)^ whereas no reports of thrombocytopenia or anemia were found. The onset of these adverse effects is variable, ranging from within a few days to after long-term use. To date, no such cases have been reported in Japan. Drug-induced blood abnormalities are known to occur via three principal mechanisms: direct drug toxicity, the effects of reactive metabolites, and immunologically mediated processes.^11)^ These mechanism effects can impact the 3 primary hematopoietic lineages—granulocytes, erythroblasts, and megakaryocytes—resulting in diverse clinical manifestations, including aplastic anemia, agranulocytosis, anemia, and thrombocytopenia. It has been reported that approximately 30% of hematologic abnormalities are caused by drug therapy, underscoring the importance of considering drug-induced causes when unexpected hematological abnormalities occur.^12)^ As there is currently no definitive test available to identify the offending drug, diagnosis remains one of exclusion. The latency period for drug-induced hematological disorders ranges from several hours to more than 1 month after exposure to drug administration. Drug-induced aplastic anemia has been reported to be latent, with an average time to symptom onset of 6.5 weeks.^12)^ Re-administration of the suspected drug is generally contraindicated due to the potential risk of severe or fatal reactions.

In the present case, the symptoms were determined to be drug-induced; however, approximately 5 months had elapsed between the initiation of tamoxifen and the onset of pancytopenia, indicating an unusually long latent period. Nonetheless, prior reports have documented hematopoietic suppression manifesting as long as late as 2 years and 5 months following the commencement of tamoxifen administration.^5)^ This variability suggests that factors such as cumulative exposure, individual susceptibility, or interactions with concomitant medications (e.g., pregabalin or prior chemotherapy) may influence the timing of onset. Although the precise mechanism remains uncertain, the possibility of a delayed immune-mediated reaction or slowly progressive bone marrow suppression may explain the extended latency observed in this case.

These potential contributions of prior chemotherapy or radiation therapy to bone marrow suppression must also be acknowledged. However, during chemotherapy (administered with granulocyte colony-stimulating factor [G-CSF] support), the only hematological abnormality observed was grade 1 anemia, and the blood counts remained within normal limits at the conclusion of treatment. Furthermore, radiation therapy for breast cancer is typically localized to the chest wall and supraclavicular region with minimal exposure to the thoracolumbar spine; thus, radiation-induced bone marrow suppression in this context is unlikely. Notably, a common feature among previously reported cases of TAM-induced cytopenia is the initiation of TAM following chemotherapy and radiation therapy.^4,5)^ This observation raises the possibility that the incidence of cytopenia may be increased in patients with prior exposure to such treatments. It is conceivable that preceding cytotoxic therapy may sensitize hematopoietic stem or progenitor cells, or may induce alterations in the bone marrow microenvironment, thereby heightening vulnerability to subsequent drug-induced suppression. The potential for additive or synergistic effects of sequential therapies should be careful.

## CONCLUSIONS

This case highlights the rare but serious side effects of TAM, including drug-induced cytopenia. Clinicians should be aware of the possibility of unexpected hematological abnormalities in patients undergoing TAM therapy, especially when associated with chemotherapy and radiation therapy.

## ACKNOWLEDGMENTS

We thank the patient and Cactus Communications for language editing.

## DECLARATIONS

### Funding

No specific funding was received for this case report.

### Authors’ contributions

A.T.: Data curation, Writing – original draft.

S.F.: Writing – review and editing.

Y.T., M.I., M.Y., K.G., T. Yamanaka, T. Yamashita, and S.Y.: Investigation.

All authors have read and approved the final manuscript.

All authors agree to be responsible for all aspects of the study.

### Availability of data and material

Not applicable.

### Ethics approval and consent to participate

Not applicable.

### Consent for publication

Written informed consent was obtained from the patient for publication of this case report.

### Competing interests

The authors declare that they have no competing interests in this case.
